# Supplementary material for: The interactome of intact mitochondria by cross-linking mass spectrometry provides evidence for coexisting respiratory supercomplexes
Source: Mol Cell Proteomics. 2017 Dec 8;17(2):216–32. doi: 10.1074/mcp.RA117.000470 (PMC5795388; doi:10.1074/mcp.RA117.000470)
Supplement: Supplemental Data [file supp_RA117.000470_133922_0_supp_23978_fzffwf.zip › spectra_annotation/mito_DR_spectra_annotation/279-1-14-1-11-1.pdf]

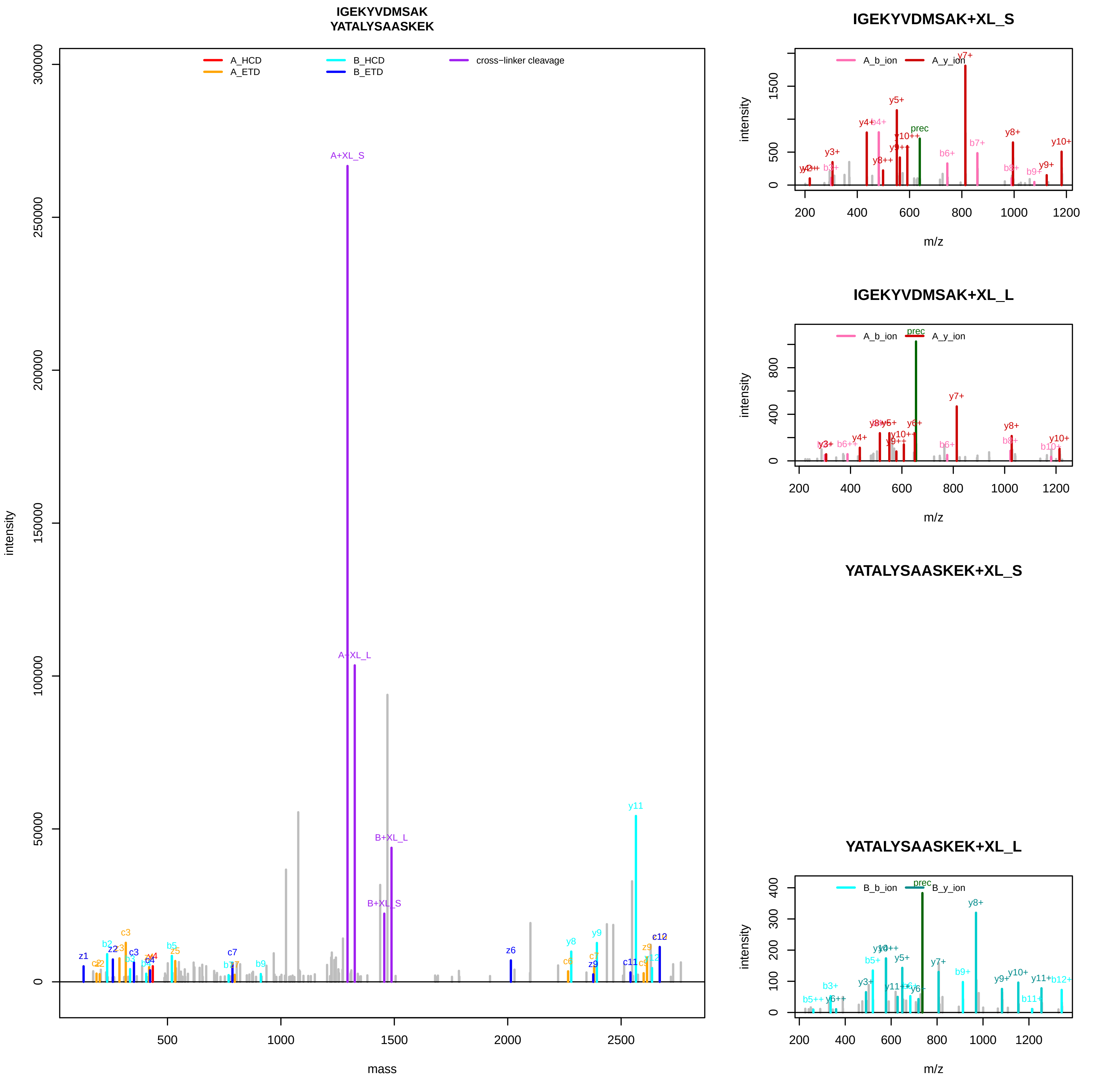

IGEKYVDMSAK
YATALYSAASKEK
A_HCD
A_ETD
B_HCD
B_ETD
cross−linker cleavage
A+XL_S
A+XL_L
B+XL_L
B+XL_S
y11
c12
y9
y8
z6
c7
b9
b5
z5
c3
b2
z2
z1
intensity
mass
IGEKYVDMSAK+XL_S
A_b_ion
A_y_ion
y7+
y5+
y4+
b4+
prec
y10++
y9++
y8++
y3+
b3+
y2++
b6+
b7+
y8+
y10+
b8+
b9+
y9+
intensity
m/z
IGEKYVDMSAK+XL_L
A_b_ion
A_y_ion
prec
y7+
y8+
y5+
y6+
y4+
y10++
y9++
b6++
y3+
b6+
y10+
b8+
b10+
intensity
m/z
YATALYSAASKEK+XL_S
YATALYSAASKEK+XL_L
B_b_ion
B_y_ion
prec
y8+
y10++
b5+
y5+
y7+
b9+
b3+
y3+
y11++
y6+
b5++
y6++
y9+
y10+
y11+
b12+
b11+
intensity
m/z
